# The Prognostic Potential of Neurokinin 1 Receptor in Breast Cancer and Its Relationship with Ki-67 Index

**DOI:** 10.1155/2022/4987912

**Published:** 2022-04-04

**Authors:** Maha S. Al-Keilani, Rana Elstaty, Mohammad A. Alqudah

**Affiliations:** ^1^Jordan University of Science and Technology, College of Pharmacy, Department of Clinical Pharmacy, P.O. Box 3030, Irbid 22110, Jordan; ^2^Jordan University of Science and Technology, College of Science and art, Department of Biotechnology and Genetic Engineering, P.O. Box 3030, Irbid 22110, Jordan; ^3^Jordan University of Science and Technology, College of Medicine, Department of Pathology and Microbiology, P.O. Box 3030, Irbid 22110, Jordan

## Abstract

**Background:**

Neurokinin 1 receptor (NK1R) is a promising biomarker and therapeutic target in breast cancer. This study was aimed at investigating the expression level of NK1R in breast cancer tissues and its relationship with proliferation index as measured by Ki-67, clinicopathological characteristics of patients, and overall survival rate.

**Methods:**

Immunohistochemical expression of NK1R and Ki-67 was measured in 164 paraffin-embedded breast cancer tissues of four molecular subtypes (42 HER2-enriched, 40 luminal A, 42 luminal B, and 40 triple negative). NK1R was scored semiquantitatively, while Ki-67 was obtained by the percentage of total number of tumor cells with nuclear staining. The optimal cutoff values for NK1R and Ki-67 were assessed by Cutoff Finder. Pearson's Chi-square (*χ*^2^) and Fisher's exact tests were used to compare the staining scores between groups. The Kaplan-Meier method with log-rank test was used for survival analysis. ANOVA and Student's *t*-test were used to compare group means.

**Results:**

A total of 164 patients were included in the study which represented females with invasive ductal carcinoma. NK1R was expressed at high levels in about 34% of investigated cases. The mean Ki-67 level was about 27% and 41.5% of sample had high Ki-67 (expression level > 22%). NK1R expression levels were associated with higher tumor grade (*p* = 0.021) and high Ki-67 (*p* = 0.012). NK1R expression negatively impacted overall survival in grade II tumors (*p* = 0.027).

**Conclusion:**

NK1R contributes to cellular proliferation and is associated with negative prognosis in breast cancer. These findings suggest the potential role of NK1R as a therapeutic target in breast cancer.

## 1. Introduction

Breast invasive ductal carcinoma also known as infiltrating ductal carcinoma is the most common form of breast cancer accounting for about 80% of breast cancer cases [1]. A general assumption is that breast tumorigenesis is a progressive process that starts with abnormal epithelial hyperproliferation followed by formation of ductal carcinoma in situ (DCIS), an early form of breast cancer that could be transformed later to an invasive carcinoma and finally to a metastatic disease [2]. Nevertheless, knowledge is lacking about which DCIS cases could transition to breast invasive ductal carcinomas due to limited information on the underlying molecular mechanisms of breast cancer progression [2, 3].

Breast cancer is a heterogeneous disease at histological, biological, pathological, and molecular levels [4]. The development of molecular analytical methods such as genetic array testing aided in the classification of breast cancer into four major molecular subtypes that predict prognosis and foretell treatment response [5]. These molecular subtypes are based on the expression level of estrogen receptor (ER), progesterone receptor (PR), and human epidermal growth factor receptor 2 (HER2) and they are as follows: luminal A, luminal B, basal-like (triple negative), and HER2-enriched tumors [5]. However, the current classification system is not sufficient to explain the high intertumor and intratumor heterogeneity of breast cancer in terms of clinical behavior and treatment response [4]. Consequently, refinement of molecular subtypes by deciphering the molecular and biological components is clinically vital and will improve the stratification of breast cancer patients for cancer therapy.

Neurokinin 1 receptor (NK1R) is a G-protein-coupled receptor and the biological mediator of the substance P tachykinin activities [6]. NK1R is under investigation as a potential biomarker and therapeutic target in cancer including breast cancer [7–11]. The NK1R was overexpressed in different types of cancers [7, 12] and was associated with several carcinogenesis processes such as angiogenesis, proliferation, apoptosis, and metastasis [7, 13, 14]. Additionally, knockdown of NK1R gene or treatment of cancer cells with NK1R antagonists such as aprepitant exerted multiple antitumoral effects [7, 9, 15–17].

Despite the extensive studies on the role of NK1R in cancer, insufficient information is available regarding its role and expression levels in breast cancer. Therefore, the aims of our study were to investigate the expression levels of NK1R in the four different molecular subtypes of breast cancer and its relationship with proliferation activity as measured by Ki-67 index and with clinicopathological parameters and overall survival rate.

## 2. Materials and Methods

### 2.1. Patients

This retrospective study was carried out in the Department of Pathology of King Abdulla University Hospital (KAUH) in Irbid, Jordan. 164 cases of breast cancer as paraffin-embedded tissues were included in this study. The cases represented female patients with invasive ductal carcinoma of stages I to IV who underwent surgical resection between 2007 and 2019 and did not receive chemotherapy or radiotherapy prior to surgery. Clinicopathological data were retrospectively collected from patients' medical charts. Surrogate clinicopathologic definitions of the molecular subtypes were used as follows:

1. HER2-enriched: ER negative, PR negative, and HER2 positive
2. Luminal A: ER positive, PR positive, HER2 negative, and low Ki-67 (<14%) [18]
3. Luminal B (Triple positive): ER positive, PR positive, HER2 positive, and any Ki-67 [18]
4. Triple negative: ER negative, PR negative, and HER2 negative

### 2.2. Tissue Microarray (TMA) Construction and TMA Slide Preparation

The cancerous areas of breast tissues were selected and marked on the identical hematoxylin and eosin (H/E) slide and sampled for TMA blocks. Eight tissue array blocks were constructed to include the entire 164 cores of interest in addition to the control cores. Brain tissues were used as positive controls for NK1R, colon tissues were used as positive controls for Ki-67, and normal breast tissues were used as negative controls.

### 2.3. Immunohistochemical Staining (IHC)

The automated Ventana Bench Mark ULTRA IHC/ISH Staining Module (Ventana Co., Tucson, AZ, USA) was used together with ultraView universal DAB (3′ diaminobenzidine) IHC detection method (Ventana Co., Tucson, AZ, USA) on the 2 *μ*m tissue sections of TMA slides.

100 *μ*l of each primary antibody for the target proteins NK1R (1: 50, ab219600; RRID is not available, Abcam, Cambridge, MA, USA) and Ki-67 (clone 30–9, prediluted, #790–4286, RRID: AB_2631262, Ventana, Tucson, AZ) were used.

### 2.4. IHC Staining Evaluation, Analysis, and Scoring

All findings were interpreted by two independent pathologists without prior knowledge of the clinical data. A light microscope (Olympus Corporation, Japan) was used to visualize the slides. Ten visual fields were selected for each slide and examined at 40x magnification, and pictures were obtained by a PC-driven digital camera (Olympus DP74, Japan).

The degree of immunostaining was examined semiquantitatively. The expression level of NK1R was based on Allred 8-unit scoring system using a combination of the proportions of positively stained cells and the intensity of the staining. Proportion of positively stained tumor cells ranged from 1% to 100%, and it was scored as follows: 0, 0% reacting cells; 1, <1% reacting cells; 2, 1%-10% reacting cells; 3, 11%-33% reacting cells; 4, 34%-66% reacting cells; and 5, > =67% reacting cells. The staining intensity scores were as follows; 0, no staining; 1, weak staining (light yellow); 2, moderate staining (yellow brown); and 3, strong staining (brown). After that the two scores were added together for a total score (TS) with eight values, cytoplasmic, nuclear, and membranous staining for NK1R was evaluated. The Ki-67 index was obtained by the percentage of total number of tumor cells with nuclear staining. All brown-stained nuclei were counted as positive regardless of staining intensity.

### 2.5. Statistical Analysis

Data were collected in an Excel database from Windows 10 (Microsoft Corporation, Redmond, WA, USA) and the SPSS statistical software system (IBM SPSS Statistics 23, USA) was used for statistical analyses. The optimal cutoff values for NK1R and Ki-67 were assessed by Cutoff Finder [19], an online application that is used to define the most relevant cutoff point for a marker to distinguish prognosis and overall survival rate in this study. Patients were categorized into two groups, those with expression level above the cutoff point as high group, otherwise as low group.

Descriptive statistics were done. Pearson's Chi-square (*χ*^2^) test or Fisher's exact test was used to compare the staining scores between groups. ANOVA and Student's *t*-test were used to compare group means. Continuous variables were presented as mean ± standard deviation, while categorical variables were presented as numbers and percentages. Survival outcomes, defined as the period from time of diagnosis to death from any cause or the last contact, were estimated with the Kaplan Meier analysis and compared between groups by log-rank test. Statistical significance was considered if *p* ≤ 0.05.

All procedures performed in the current study were approved by the Institutional Review Board at Jordan University of Science and Technology (28/116/2018) in accordance with the 1964 Helsinki Declaration and its later amendments.

## 3. Results

### 3.1. Clinical and Pathological Features of Breast Cancer Patients

A total of 164 patients were included in the study. All cases represented females with invasive ductal carcinoma. The average age of patients was 51.35 ± 11.2 years (extremes: 28-82). The detailed patients and tumor characteristics are displayed in Table 1. Regarding tumor volume it was calculated using the ellipsoid model formula: tumor volume (cubic centimeter) = *π*/6 (*a* × *b* × *c*), where *a*, *b*, and *c* represent three perpendicular diameters.

**Figure 1:**
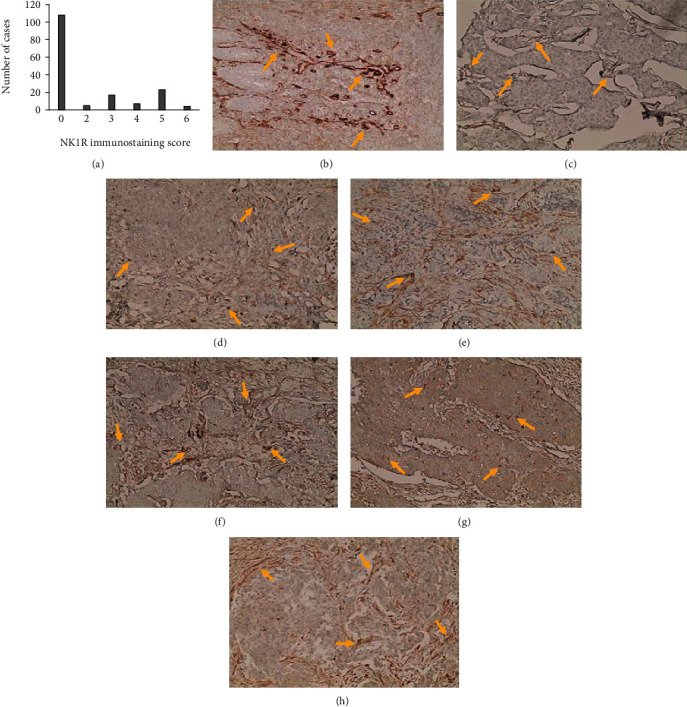
Immunohistochemical staining of NK1R in breast cancer. (a) The scoring of immunohistochemical staining for NK1R. (b) Positive control (brain tissue). (c–h) NK1R expression level (mainly in the cytoplasm). (c and d) Low expression of NK1R and (e–h) high expression for NK1R. Abbreviation: NK1R; neurokinin 1 receptor.

The expressions of NK1R and Ki-67 in invasive ductal carcinoma of the breast were detected by IHC. Allred 8-unit semiquantitative scoring of NK1R expression was used. A representative graph for the frequency of the eight total scores is shown in Figure 1(a).

One hundred and eight samples (65.9%) negatively expressed NK1R (TS = 0), 3% had TS of 2, 10.4% had TS of 3, 4.3% had TS of 4, 14.0% had TS of 5, and 2.4% had a total score of 6. No cases had TS of 7 or 8. The mean Ki-67 level was about 27%.

**Figure 2:**
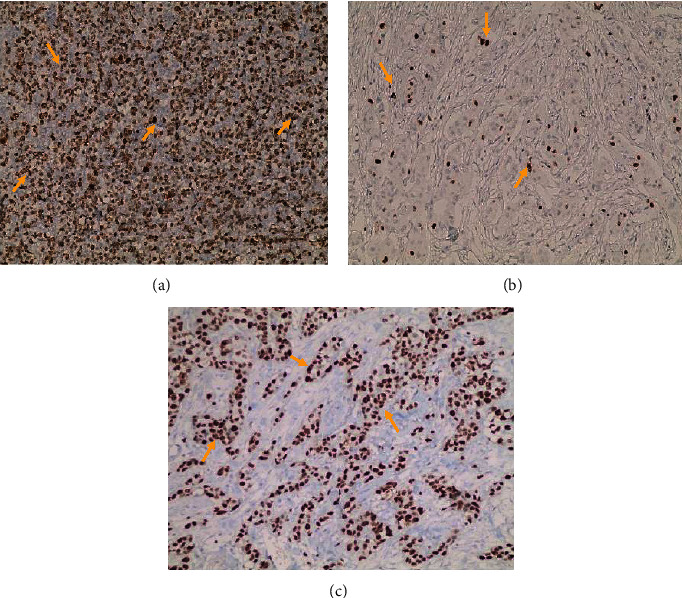
Immunohistochemical staining of Ki-67 in breast cancer and positive control; manifested mainly in the nucleus. (a) Positive control (colon tissues), (b) low Ki-67 expression, and (c) high Ki-67 expression level.

Using the online Cutoff Finder, the optimal cutoff values for NK1R and Ki-67 were a total score of 1 for NK1R and expression level of 22% for Ki-67. Consequently, we defined patients with NK1R TS > 1 or Ki-67 expression > 22% as the NK1R or Ki-67 high expression groups. There were 108 (65.9%) patients with NK1R TS ≤ 1 and 56 (34.1%) patients with NK1R TS > 1. There were 96 (54.5%) patients with Ki − 67 ≤ 22% and 68 (41.5%) patients with Ki − 67 > 22%. Representative IHC images for NK1R and Ki-67 are shown in Figures 1(b)–1(h) and 2(a)–2(c), respectively.

### 3.2. Association between NK1R and Clinicopathological Parameters

As revealed in Table 2, there were no associations between NK1R expression level and age, breast cancer molecular subtype, tumor volume, TNM stage, pT stage, pN stage, distant metastasis, axillary lymph node metastasis or lymphovascular invasion, ER, PR and HER2 statuses, DCIS history, family history, or type of therapy. A significant association was found between NK1R expression level and tumor grade (*χ*^2^ = 7.212, *p* = 0.021). Forty-four (78.6%) of 56 patients with high NK1R expression had grade III tumors and the remaining had grade II tumors. Additionally, low NK1R expression was associated with low Ki-67 index (*χ*^2^ = 6.763, *p* = 0.012); seventy-one (65.7%) of 108 patients with low NK1R expression had low Ki-67 expression.

### 3.3. Association between NK1R Expression and Survival Outcomes

**Figure 3:**
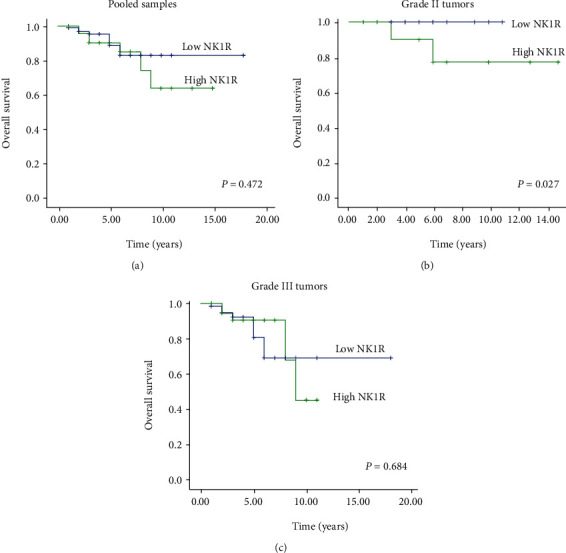
Overall survival analysis based on NK1R expression in pooled samples and different tumor grades. (a) Overall survival based on NK1R expression in pooled samples. (b) Overall survival based on NK1R expression in grade II tumors. (c) Overall survival based on NK1R expression in grade III tumors. Abbreviation: NK1R; neurokinin 1 receptor. Note: NK1R was not expressed in the investigated grade I tumors.

**Figure 4:**
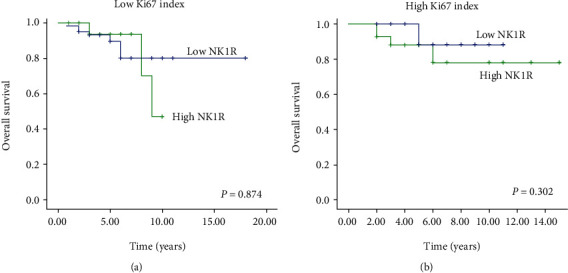
Overall survival analysis based on NK1R expression and Ki-67 index. (a) Overall survival based on NK1R expression in tumors with low Ki-67 index. (b) Overall survival based on NK1R expression in tumors with high Ki-67 index. Abbreviation: NK1R; neurokinin 1 receptor.

We next investigated the effect of NK1R expression level on overall survival. There was no significant difference between low and high expression groups when all cases were pooled together (Figure 3(a), *p* = 0.472). However, since NK1R expression was associated with tumor grade, we further investigated its effect on overall survival in those groups. A negative impact of NK1R expression was seen in grade II tumors (Figure 3(b), *p* = 0.027) but not in grade III tumors (Figure 3(c), *p* = 0.684). We also investigated the impact of NK1R expression on overall survival by Ki-67 index; however, there was no significant difference between the groups (*p* > 0.05, Figure 4).

## 4. Discussion

NK1R could be a useful marker that provides clues about prognosis and response to therapy and may represent a new therapeutic target in breast cancer. Few studies are available that explore NK1R expression and investigate its prognostic value in breast cancer [12, 20–22]. This study retrospectively evaluated the expression of NK1R in 164 breast invasive ductal carcinoma cases from the four molecular subtypes. Additionally, the relationships between the NK1R expression and cell proliferation as measured by Ki-67 index and with clinicopathological parameters and overall survival rate were tested.

We observed that NK1R was high in about 34% of cases. Significant associations were found between NK1R expression level and tumor grade (*p* = 0.021) and Ki-67 index (*p* = 0.012). NK1R expression was found to be higher in grade III tumors (78.6%) when compared with grade II (21.4%) and grade I (0.0%). In a study by Garcia-Recio et al., NK1R was positively expressed in 94% of the analyzed cases (318 samples) [21]. A study by Davoodian et al. showed that NK1R expression was positive in all the thirty cases analyzed [22], and it was prominent in about 88% of analyzed tissues in another study by Huang et al. [23]. The variation in the expression rate could be due to the differences in the histological type of breast cancer cases analyzed, besides the differences in the sample size. Another explanation is the presence of two isoforms for NK1R, the full length (NK1R-FL) and the truncated (NK1R-Tr). The latter is characterized by the absence of 96 residues in its cytoplasmic end, which may reduce the efficiency with respect to desensitization and internalization [24]. It is still unclear how NK1-FL or NK1-Tr is functionally linked to tumorigenesis and their specific biological functions in tumor formation and progression. Nevertheless, previous studies demonstrated that NK1R-FL expression levels were obviously reduced in breast cancer cell lines and tumor tissues and significantly overexpressed in normal breast tissues, whereas NK1R-Tr form was highly expressed in breast cancer cells and tissues [25, 26]. High gene expression of NK1R-Tr significantly associated with TNM stage, ER, PR statuses, and Ki-67 expression, whereas NK1R-FL was not associated with any of the clinicopathological variables except for lymph node status [25]. While in our study we applied a nonspecific primary antibody that binds to the extracellular domain of the receptor thus detecting both isoforms, future studies should focus on investigating the two isoforms to identify their differential expression and prognostic value in breast cancer patients.

In concordance with our results, one previous study revealed a significant association between NK1R expression and tumor grade, but differential expression among the three grades was not reported in that study [23]. In another study, high NK1R expression was evident in grade II and grade III breast cancer tissues but analysis for statistically significant difference between grades was not performed [12]. These findings indicate a prognostic value of NK1R in breast cancer patients.

To the best of our knowledge, this is the first study to report a positive association between NK1R immunohistochemical expression and Ki-67 index in breast cancer. A similar finding was reported in oral squamous cell carcinoma [13] and malignant odontogenic tumors [27]. Accordingly, treatment of animal models of brain tumor, pancreatic cancer, and hepatoblastoma with NK1R antagonists resulted in a significant reduction in Ki-67 positive cells [28–30], therefore supporting the previous evidence of an oncogenic effect of NK1R through enhancing tumor cell proliferation [16, 31].

Our survival analysis demonstrated a clear negative impact of NK1R expression level on overall survival of patients with grade II tumor. This result supports the potential role of NK1R as a therapeutic target in breast cancer; thus, future studies must focus on identifying the underlying mechanisms of the role of this protein in breast cancer.

As a conclusion, in this study, we were able to identify the expression level of NK1R in breast invasive ductal carcinomas of the four molecular subtypes: HER2-enriched, luminal A, luminal B, and triple negative tumors. High NK1R expression level was associated with high tumor grade and high Ki-67 index. Moreover, NK1R expression had a negative impact on overall survival in grade II tumors. From the results of this study, we can propose a role of NK1R as negative prognostic marker in breast cancer. Nevertheless, analysis of larger cohort of patients in more complex studies is required, while focusing on the application of inexpensive and practical methods like IHC. Moreover, future studies must focus on investigating the gene expression of the target proteins to allow for biomarker and gene signature discovery.

## Figures and Tables

**Table 1:** Demographic, clinical, and histopathological characteristics of 164 breast cancer patients.

Variable	Total (*n*%)
*Age (years)*	
Mean ± SD	51.35 ± 11.2
Range	28-82
*Molecular subtype*	
HER2-enriched	42 (25.6)
Luminal A	40 (24.4)
Luminal B	42 (25.6)
Triple negative	40 (24.4)
*Grade*	
I	8 (4.9)
II	46 (28)
III	110 (67.1)
*Tumor volume (*cm^3^)	
Mean ± SD	38.97 ± 67.41
Range	0.18-571.77
*TNM stage*	
I	7 (4.3)
II	45 (27.4)
III	56 (34.1)
IV	54 (32.9)
Missing	2 (1.2)
*pT stage*	
T1	13 (7.9)
T2	88 (53.7)
T3	47 (28.7)
T4	16 (9.8)
*pN stage*	
N0	44 (26.8)
N1	39 (23.8)
N2	33 (20.1)
N3	43 (26.2)
Missing	5 (3.0)
*Distant metastasis*	
M0	108 (65.9)
M1	54 (32.9)
Missing	2 (1.2)
*Axillary lymph node metastasis*	
Negative	45 (27.4)
Positive	117 (71.3)
Missing	2 (1.2)
*Lymphovascular invasion*	
Negative	31 (18.9)
Positive	94 (57.3)
Missing	39 (23.8)
*ER status*	
Negative	82 (50)
Positive	82 (50)
*PR status*	
Negative	82 (50)
Positive	82 (50)
*HER2 status*	
Negative	80 (48.8)
Positive	84 (51.2)
*DCIS history*	
Absent	27 (16.5)
Present	113 (68.9)
Missing	24 (14.6)
*Family history*	
No	106 (64.6)
Yes	29 (17.7)
Missing	29 (17.7)
*Adjuvant chemotherapy*	
No	4 (2.4)
Yes	120 (73.2)
Missing	40 (24.4)
*Adjuvant radiotherapy*	
No	75 (45.7)
Yes	49 (29.9)
Missing	40 (24.4)
*Hormonal therapy*	
No	62 (37.8)
Yes	62 (37.8)
Missing	40 (24.4)
*Immunotherapy*	
No	118 (72.0)
Yes	6 (3.7)
Missing	40 (24.4)
*Ki-67 index*	
Low	96 (58.5)
High	68 (41.5)
*NK1R*	
Low	108 (65.9)
High	56 (34.1)

DCIS: ductal carcinoma in situ; ER: estrogen receptor; PR: progesterone receptor; NK1R: neurokinin 1 receptor.

**Table 2:** Association between neurokinin 1 receptor expression level and clinicopathological parameters.

Parameters	Low or no expression	High expression	*p*
	Percentage (%)/mean ± SD	Percentage (%)/mean ± SD	
Age (years)	51.94 ± 11.61	50.21 ± 10.41	0.350
*Molecular subtype*			0.493
HER2-enriched	26 (24.1)	16 (28.6)	
Luminal A	30 (27.8)	10 (17.9)	
Luminal B	28 (25.9)	14 (25.0)	
Triple negative	24 (22.2)	16 (28.6)	
*Grade*			**0.021**
I	8 (7.4)	0 (0.0)	
II	34 (31.5)	12 (21.4)	
III	66 (61.1)	44 (78.6)	
Tumor volume (cm^3^)	33.74 ± 52.14	49.04 ± 89.58	0.169
*TNM stage*			0.427
I	4 (3.7)	3 (5.6)	
II	34 (31.8)	11 (20.4)	
III	34 (31.8)	22 (40.7)	
IV	35 (32.7)	18 (33.3)	
*pT stage*			0.628
T1	8 (7.4)	5 (8.9)	
T2	60 (55.6)	28 (50.0)	
T3	28 (25.9)	19 (33.9)	
T4	12 (11.1)	4 (7.1)	
*pN stage*			0.136
N0	27 (25.5)	17 (32.1)	
N1	32 (30.2)	7 (13.2)	
N2	20 (18.9)	13 (24.5)	
N3	27 (25.5)	16 (30.2)	
*Distant metastasis*			0.860
M0	72 (67.3)	35 (64.8)	
M1	35 (32.7)	19 (35.2)	
*Axillary lymph node metastasis*			0.356
Negative	27 (25.2)	18 (32.7)	
Positive	80 (74.8)	37 (67.3)	
*Lymphovascular invasion*			1.000
Negative	36 (34.0)	17 (32.7)	
Positive	70 (66.0)	35 (67.3)	
*ER status*			0.249
Negative	50 (46.3)	32 (57.1)	
Positive	58 (53.7)	24 (42.9)	
*PR status*			0.249
Negative	50 (46.3)	32 (57.1)	
Positive	58 (53.7)	24 (42.9)	
*HER2 status*			0.742
Negative	54 (50.0)	26 (46.4)	
Positive	54 (50.0)	30 (53.6)	
*DCIS history*			0.331
Absent	23 (21.7)	16 (29.6)	
Present	83 (78.3)	38 (70.4)	
*Family history*			0.270
No	74 (81.3)	32 (72.7)	
Yes	17 (18.7)	12 (27.3)	
*Adjuvant chemotherapy*			0.598
No	2 (2.4)	2 (4.9)	
Yes	81 (97.6)	39 (95.1)	
*Adjuvant radiotherapy*			0.699
No	49 (59.0)	26 (63.4)	
Yes	34 (41.0)	15 (36.6)	
*Hormonal therapy*			0.252
No	38 (45.8)	24 (58.5)	
Yes	45 (54.2)	17 (41.5)	
*Immunotherapy*			1.000
No	79 (95.2)	39 (95.1)	
Yes	4 (4.8)	2 (4.9)	
*Ki-67*			**0.012**
Low	71 (65.7)	25 (44.6)	
High	37 (34.3)	31 (55.4)	

ER: estrogen receptor; PR: progesterone receptor; DCIS: ductal carcinoma in situ. *p* values in bold are those <0.05.

## Data Availability

The datasets generated during and/or analyzed during the current study are available from the corresponding author on reasonable request.

## Abbreviations

NK1R: Neurokinin 1 receptor
HER2: Human epidermal growth factor receptor 2
DCIS: Ductal carcinoma in situ
ER: Estrogen receptor
PR: Progesterone receptor
TMA: Tissue microarray
IHC: Immunohistochemistry.
